# Complete and long-lasting response to immunotherapy: A case report of urothelial cancer

**DOI:** 10.1097/MD.0000000000028940

**Published:** 2022-06-17

**Authors:** Diogo J. Silva, Alexandra Mesquita

**Affiliations:** Medical Oncology Department, Local Health Unity Matosinhos–Hospital Pedro Hispano, Porto, Portugal.

**Keywords:** case report, cisplatin ineligible, immunotherapy, urothelial cancer

## Abstract

**Introduction:**

Bladder cancer is the tenth most common cancer worldwide, with Europe having the highest incidence rates. Regarding the treatment of metastatic disease, first-line treatment for fit patients is cisplatin-containing combination chemotherapy. However, a significant percentage of patients are ineligible for platinum-based chemotherapy, or progress under these regimens. Recently, immune checkpoint blockade has become a treatment option for this group of patients. In this report, we present the case of a male patient diagnosed with metastatic bladder cancer who did not tolerate cisplatin-containing chemotherapy and achieved complete response after treatment with pembrolizumab.

**Patient concerns:**

A 58 years-old Caucasian man with a medical history of high-grade urothelial carcinoma pT3bN0R0 under a watchful waiting strategy for 6 months presented to the Medical Oncology appointment with two axillary and cervical adenopathies.

**Diagnosis:**

Cervicothoracoabdominal computed tomography confirmed the presence of two large necrotic lymphadenopathies in the cervical and axillary lymphatic chains, and bone scintigraphy revealed dorsal (D11) and lumbar (L5) metastatic lesions. Ultrasonography-guided biopsy of the axillary nodule revealed the presence of metastatic tissue of primary urothelial origin.

**Interventions:**

The patient was initiated on a palliative chemotherapy regimen of carboplatin area under the curve 5 plus gemcitabine (1000 mg/m^2^). During the first cycle of chemotherapy, acute kidney failure akin 2 developed due to nonobstructive toxic acute tubular necrosis with progressive deterioration of kidney function. Therefore, palliative chemotherapy with carboplatin plus gemcitabine was changed to 200 mg of pembrolizumab every 21 days.

**Outcomes:**

Overal survival of 57 months with an immune complete response according to the immune Response Evaluation Criteria in Solid Tumours criteria and an excellent quality of life.

**Conclusion:**

This case illustrates that second-line therapy with ICIs (pembrolizumab or atezolizumab) has favourable results in achieving an immune complete response after intolerance to cisplatin-based regimens. ICIs provide durable responses that improve overall survival and quality of life.

## 1 Introduction

Bladder cancer is the tenth most common cancer worldwide, with Europe having the highest incidence rates. European patients have an age-standardized incidence rate of 17.7 for males and 3.5 for females, with 70% being older than 65 years.^[1]^ Tobacco smoking is the main risk factor, and smokers have a 2.5 times higher risk of bladder cancer than non-smokers. Although cigarette smoking is the most common risk factor, occupational exposure, such as industries involving paint, metal, and oil products, also has a higher risk. Radical cystectomy with lymph node dissection is the treatment of choice for localised disease. For metastatic disease, the first-line treatment for fit patients is cisplatin-containing combination chemotherapy.^[2]^ A significant percentage of patients are ineligible for platinum-based chemotherapy or progress under these regimens. Recently, immune checkpoint blockade has become a treatment option for this group of patients.^[3]^

## 2 Case report

A 58 years-old Caucasian man with no relevant past medical history presented to the emergency department complaining of gross sporadic haematuria that had evolved during the last six months. Abdominopelvic computed tomography revealed an extensive tumoural bladder lesion without signs of metastatic spread-cT2N0M0. Radical cystoprostatectomy with ureteroileostomy is complicated by anastomosis dehiscence and the need for derivative ileostomy. Histopathological analysis revealed a high-grade urothelial carcinoma, pT3bN0R0. The surgical wound was closed with negative pressure dressing, and the patient was discharged to a multidisciplinary team appointment after 11 days. After multidisciplinary team assessment, adjuvant chemotherapy was administered. However, owing to inflammatory signs and exudative drainage from the surgical wound, chemotherapy was postponed until healing, and the time for adjuvant chemotherapy benefit was outdated. After case revision during the multidisciplinary team appointment, the patient underwent a watchful waiting strategy approach in July/2017. In November 2017, the patient presented to the emergency department complaining of pain and unilateral swelling of the left lower limb for 72 hours.

**Figure 1:**
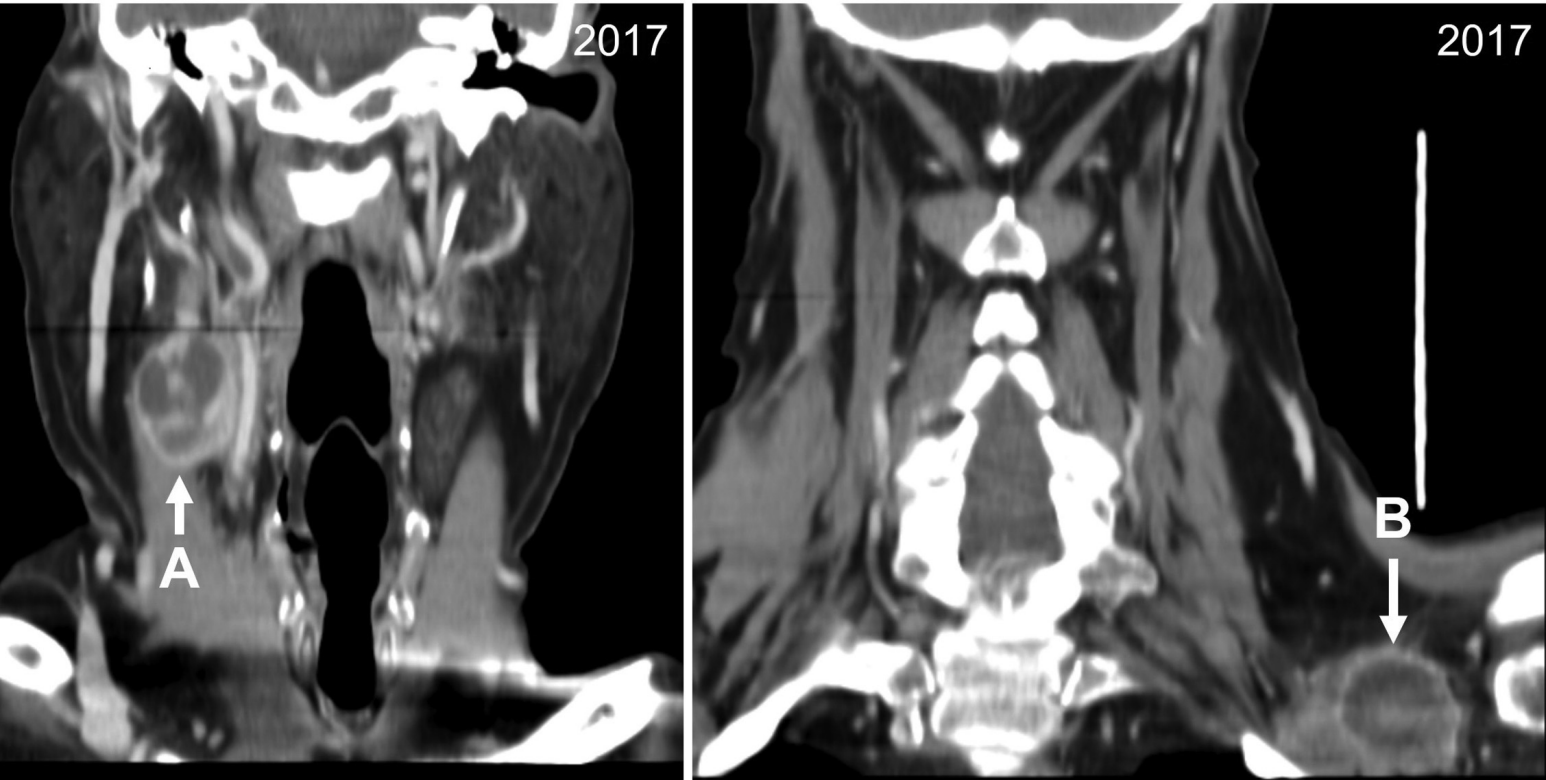
Cervical and axillary metastatic lymphadenopathies in 2017.

During the physical examination, two axillary and cervical adenopathies, along with inflammatory signs and tenderness of the lower left limb, were noted. Cervicothoracoabdominal computed tomography confirmed the presence of two large necrotic lymphadenopathies in the cervical and axillary lymphatic chains without further evidence of metastatic disease (Fig. 1). Ultrasonography-guided biopsy of the axillary nodule revealed the presence of metastatic tissue of primary urothelial origin (CK7 and p53 positive; cytokeratin 20 negative). Furthermore, bone scintigraphy revealed dorsal (D11) and lumbar (L5) metastatic lesions. Facing evidence of stage IV urothelial carcinoma with lymphatic and bone metastases, palliative chemotherapy was initiated.

According to the physician's choice, the patient was initiated on a palliative chemotherapy regimen using carboplatin area under the curve 5 plus gemcitabine 1000 mg/m^2^ in December/2017. During the first cycle of chemotherapy, the patient developed acute kidney failure akin 2 due to nonobstructive toxic acute tubular necrosis with progressive improvement after inpatient admission for intravenous fluid therapy. Although renal function improved, creatinine clearance was only 30 ml/minute/1.73 m^2^ at this time. Therefore, palliative chemotherapy with carboplatin plus gemcitabine was changed to 200 mg in January/2018. After two cycles of pembrolizumab, new renal impairment with acute kidney lesion akin to 3, and inpatient admission for further care. Due to the possibility of autoimmune nephritis to pembrolizumab, empirical therapy with corticosteroid was initiated, and urinalysis plus renal ultrasonography was performed after observation by a nephrologist. Normal urinalysis with renal ultrasonography showed bilateral cortical hyperechogenicity and right hydroureteronephrosis. Renopelvic computed tomography revealed a small stone (8 mm) on the right ureteroileostomy, explaining sudden renal function degradation and leading to right percutaneous nephrostomy. Given the good response to pembrolizumab with resolution of the right cervical adenopathy after the first cycle of treatment, no changes in therapy were performed. After seven cycles of pembrolizumab, bone scintigraphy showed persistence of dorsal and lumbar bone metastases previously known with evidence of a new bone lesion on the eleventh right rib. Re-evaluation thoracoabdominopelvic computed tomography showed an immune partial response according to the immune Response Evaluation Criteria in Solid Tumours (iRECIST) criteria. Twelve cycles of pembrolizumab were completed in December/2018 with reassessment bone scintigraphy revealing stable bone disease.

**Figure 2:**
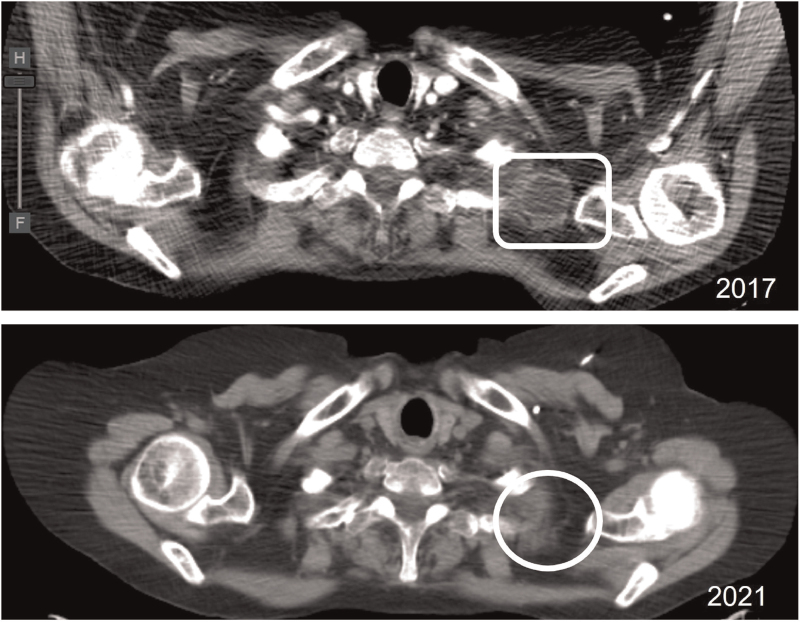
Complete response of left axillary metastatic lymphadenopathy after immunotherapy with Pembrolizumab.

Considering the progressive deterioration of renal function and stable disease after pembrolizumab treatment, a watchful watching strategy was adopted. In December/2020, a new assessment of bone disease by bone scintigraphy showed no evidence of bone lesions, with thoraco-abdominopelvic computed tomography showing immune complete response (iCR) according to the iRECIST criteria (Fig. 2). With an overall survival of 44 months, the patient remains asymptomatic and has an excellent quality of life.

## 3 Discussion

At diagnosis, 75% of patients present with non-muscle-invasive bladder cancer and 25% with muscle-invasive bladder cancer or metastatic disease.^[4]^ Metastatic disease is incurable with platinum-based chemotherapy such as metrothexate, vinblastine, doxorubicin, and cisplatin or gemcitabine-cisplatin, which is the standard of care for advanced urothelial cancer (UC). Having similar survival outcomes, the better toxicity profile and lower death rate led to a wider acceptance of gemcitabine plus cisplatin as standard first-line therapy.^[5]^ Despite being the optimal treatment, cisplatin is scarcely tolerated and the European Organisation for Research and Treatment of Cancer 30986 trial favoured carboplatin plus gemcitabine for patients who cannot tolerate cisplatin.^[6]^ Overall, cisplatin-based chemotherapy use has been limited by toxicity, rarely achieving complete and durable remissions in metastatic UC. In recent years, molecular profiling of muscle-invasive bladder cancer has allowed the definition of five different subtypes: luminopapillary, luminal, basal-squamous, luminopapillary, and neuronal. Despite the unclear prognostic and clinical implications of these molecular findings, epidermal growth factor receptor alterations, programmed cell death ligand 1 (PD-L1) status, DNA damage response, and repair gene alterations may guide treatment management in cases where platinum-based therapy fails or cannot be tolerated.^[4]^ The definition and characterisation of immune checkpoints allowed the development of several immunotherapy agents and changed the paradigm of advanced UC after platinum-based chemotherapy. Programmed cell death 1 is a T cell receptor that binds to PD-L1 on the surface of normal cells and limits the immune response. Expression of PD-L1 by cancer cells is a mechanism to evade the immune system but may also be associated with increased response to treatment. The FDA has approved several antibodies against Programmed cell death 1 immune checkpoint inhibitors (ICI) (nivolumab and pembrolizumab) or PD-L1 ligand (atezolizumab) as first-and second-line therapies for advanced UC.^[3]^ Regarding first-line use, ICIs are limited to patients with locally advanced or metastatic UC not eligible for cisplatin-based chemotherapy, whose expression of PD-L1 is positive or ineligible for any platinum-based regimen regardless of PD-L1 status.^[7]^

As second-line therapy, IMvigor 210 (a phase II trial) granted approval for the use of atezolizumab for locally advanced or metastatic UC refractory to platinum-containing chemotherapy in 2016. The results of this phase II trial indicated a higher clinical activity favouring PD-L1 inhibition over classic systemic chemotherapies, with a better safety profile. This was considered the first improvement in standard-of-care second-line therapies and allowed the approval of pembrolizumab in 2017 by the KEYNOTE-045 phase III trial for patients with locally advanced or metastatic UC showing disease progression during or after platinum-containing chemotherapy.^[8]^ Within the pembrolizumab cohort, patients had a higher ORR (21% vs. 14%) and statistically significant improved overall survival with 27% death risk reduction (10.2 vs 7.4 moths of OS). The effect of pembrolizumab on OS was independent of PD-L1 expression if the CPS score was ≥ 10%. Additionally, long-term follow-up presented at the 2018 American Society of Clinical Oncology GenitoUrinary meeting stated the maintenance of survival benefit for pembrolizumab at 24 months.

Looking in detail at second-line monotherapy with pembrolizumab, the case reported illustrates some of the important aspects when dealing with locally advanced or metastatic UC treated with immunotherapy:

## 4 Imune complete response (iCR)

The first trial documenting iCR after monotherapy with pembrolizumab was the Keynote-052 trial, with 5% of cisplatin-ineligible patients achieving iCR (n = 370).^[7]^ on these results, the Keynote-012 trial, a non-randomised open-label phase 1b basket trial for locally advanced or metastatic UC, documented the achievement of complete immune response in 3 (11%) out of 27 patients.^[9]^ Moreover, the recent results of two-year follow-up after the Keynote-045 trial showed that a complete immune response was achieved in 9.3% of patients.^[10]^

## 5 Durable response

Long-lasting response after immunotherapy reflect the mechanism of action of ICI) and its modulation of lymphocytes T. ICIs stimulate cancer-specific immune response by restoring active infiltrates of T cells, creating new patterns of response, challenging the old idea of treating until progression in advanced or metastatic cancer.^[11]^ Regarding durable response, no clear definition exists, and median progression free survival time differs from trial to trial. In Keynote-045, the median duration of response was not established, but 68% of the patients were expected to have a response duration of at least 12 months. After 13 months of follow-up, 26% (n = 7/27) of patients achieved an overall response of 11% (n = 3/27) presenting iCR in the Keynote-012 trial. Analysis of the Keynote-045 results showed that 78% of the patients had a minimal duration of response lasting at least 6 months.

## 6 Median time to response

During the first cycle of pembrolizumab, an early response with resolution of cervical metastases was observed during the first month of treatment, and iCR was achieved 6 months after the suspension of pembrolizumab. To our knowledge, such an early response is uncommon, with a median time to response of approximately 2 months in various trials.^[7,8,9]^

## 7 Imunne-specific related response criteria

As stated before, response patterns of solid tumours to immunotherapy are substantially different from those observed with cytotoxic agents and molecularly-target agents.^[11]^ Durable responses, pseudo-progression, hyper-progression and dissociated responses are four of the new patterns observed during immunotherapy treatment. No clear definition for durable responses exists, whereas pseudo-progression is defined as the radiographic or clinical appearance of treatment failure due to transient changes with an evolution toward response and clinical recovery. Hyperprogression consists of a seemingly faster growth of disease after the initiation of immunotherapy.^[11,12]^

These different response patterns have practical clinical implications, which urge the need for new immune-related response criteria such as immune-related response criteria (immune-related response criteria), irRECIST (immune-related RECIST), and iRECIST (immunotherapy RECIST).^[12]^ iRECIST guidelines were created by the RECIST working group based on the Response Evaluation Criteria in Solid Tumours version 1.1. Tumour response assessed by iRECIST guidelines is subdivided into iCR, immune partial response, unconfirmed progressive disease, or confirmed progressive disease. However, the iRECIST is a consensus-based guideline that has not yet been validated.^[13]^

## 8 Conclusion

This case reinforces the possibility of achieving iCR using monotherapy with ICI as second-line therapy after intolerance to cisplatin-based regimens. Moreover, it highlights the long-lasting response after immunotherapy, with an overall survival of 57 months and excellent quality of life.

## Author contributions

AM was the medical oncologist responsible for the chemotherapy treatment, data assessment, and manuscript review. DJS was responsible for the literature review, data assessment, manuscript writing, and reviewing.

**Conceptualization:** Diogo Silva.

**Data curation:** Alexandra Mesquita, Diogo Silva.

**Formal analysis:** Alexandra Mesquita, Diogo Silva.

**Writing – original draft:** Diogo Silva.

**Writing – review & editing:** Diogo Silva.
